# Origin and fate of methane in the Eastern Tropical North Pacific oxygen minimum zone

**DOI:** 10.1038/ismej.2017.6

**Published:** 2017-02-28

**Authors:** Panagiota-Myrsini Chronopoulou, Felicity Shelley, William J Pritchard, Susanna T Maanoja, Mark Trimmer

**Affiliations:** 1 School of Biological and Chemical Sciences, Queen Mary University of London, London, UK

## Abstract

Oxygen minimum zones (OMZs) contain the largest pools of oceanic methane but its origin and fate are poorly understood. High-resolution (<15 m) water column profiles revealed a 300 m thick layer of elevated methane (20–105 nM) in the anoxic core of the largest OMZ, the Eastern Tropical North Pacific. Sediment core incubations identified a clear benthic methane source where the OMZ meets the continental shelf, between 350 and 650 m, with the flux reflecting the concentration of methane in the overlying anoxic water. Further incubations characterised a methanogenic potential in the presence of both porewater sulphate and nitrate of up to 88 nmol g^−1^day^−1^ in the sediment surface layer. In these methane-producing sediments, the majority (85%) of methyl coenzyme M reductase alpha subunit (*mcrA*) gene sequences clustered with Methanosarcinaceae (⩾96% similarity to *Methanococcoides* sp.), a family capable of performing non-competitive methanogenesis. Incubations with ^13^C-CH_4_ showed potential for both aerobic and anaerobic methane oxidation in the waters within and above the OMZ. Both aerobic and anaerobic methane oxidation is corroborated by the presence of particulate methane monooxygenase (*pmoA*) gene sequences, related to type I methanotrophs and the lineage of *Candidatus* Methylomirabilis oxyfera, known to perform nitrite-dependent anaerobic methane oxidation (N-DAMO), respectively.

## Introduction

Methane is the most abundant hydrocarbon in the atmosphere and a potent greenhouse gas, which has contributed ~20% to the Earth's warming since pre-industrial times (Lashof and Ahuja, 1990; Reeburgh, 2007; Kirschke *et al.*, 2013). The marine environment encompasses large reservoirs of methane (Zhang *et al.*, 2011; Pack *et al.*, 2015), particularly in oxygen minimum zones (OMZs). Here, oxygen is consumed faster than it is resupplied, resulting in a layer of hypoxic waters surrounding a functionally anoxic core (Thamdrup *et al.*, 2012), where methane accumulates (Wright *et al.*, 2012). Under a warming climate, the dissolution of oxygen in seawater will decrease, whereas its consumption through respiration will likely increase (Vázquez-Domínguez *et al.*, 2007) and thermal stratification could become more intense. Together, these biotic and abiotic changes will thicken OMZs moving these large methane pools closer to the zone of atmospheric exchange (Stramma *et al.*, 2008; Keeling *et al.*, 2010; Helm *et al.*, 2011).

Marine methanogenesis, which produces 0.7–1.4Tg each year (Krüger *et al.*, 2005), forms an essential link in the carbon cycle, preventing the long-term burial of carbon in the sediments by mineralising it and returning it to the water in gaseous form (Ferry and Lessner, 2008). The thermodynamics of organic matter oxidation dictate that sulphate reduction and methanogenesis should be mutually exclusive reactions. Although the clear spatial partitioning of these two microbial processes has been widely observed in marine sediments (Martens and Berner, 1977; Whiticar, 2002; Reeburgh, 2007), non-competitive methanogenesis can co-occur with other anaerobic processes (Valentine, 2011). Non-competitive methanogenisis disproportionates methylated substrates (for example, methyl amine, methane thiols and methanol) to yield methane and carbon dioxide, and since its discovery in the early 1980s (Oremland *et al.*, 1982), it has been found to occur in all major oceans (D'Hondt *et al.*, 2002; Mitterer, 2010; Valentine, 2011). However, relatively little scientific attention has focused on microbial methanogenesis compared with that around gas hydrates and cold seeps (Shakhova *et al.*, 2005; Valentine, 2011; Boetius and Wenzhofer, 2013).

The majority of methane produced in marine sediments is thought to be oxidised anaerobically, limiting its flux to the overlying water (Knittel and Boetius, 2009). Any methane leaking into the water column may still be oxidised, by pelagic aerobic or anaerobic bacteria, which form a final barrier preventing its escape to the atmosphere (Reeburgh *et al.*, 1991; Blumenberg *et al.*, 2007; Kessler *et al.*, 2011; Heintz *et al.*, 2012).

We focused on locating the origin of methane in the Eastern Tropical North Pacific (ETNP), between 70 and 720 km off the Guatemalan coast. The ETNP OMZ is both the world's largest OMZ (Paulmier and Ruiz-Pino, 2009) and the largest reservoir of oceanic methane (Sansone *et al.*, 2001, 2004; Reeburgh, 2007; Naqvi *et al.*, 2010). Here, the methane is thought to be formed by a combination of decomposing sinking organic matter and coastal or benthic sources but neither have been directly measured (Sansone *et al.*, 2001, 2004). Porewater and bottom-water methane concentrations along with stable isotope ratio data suggested the sediments were the source of the pelagic methane and the flux was greatest where the anoxic core of the OMZ touched the sediment (western Mexican margin, Sansone *et al.* (2004)). Although these studies offer useful insights, there are no direct measurements of sediment methanogenesis or methane efflux in a marine OMZ.

Pelagic methane oxidation in marine environments is a rarely quantified process but on the margins of an OMZ, where methane intersects traces of oxygen, it could be a significant process. Published rates span ~0.001–10 nmol l^−1^day^−1^ and all studies used either ^3^H-CH_4_ or LL-^14^C as a tracer (Mau *et al.*, 2013). The only study to have successfully measured methane oxidation in the ETNP OMZ (Pack *et al.*, 2015) found exceptionally slow rates (0.000034–4 nmol l^−1^day^−1^), which could explain how the methane, if of benthic origin, can be sustained hundreds of kilometres offshore.

We used high-resolution water column profiles to show that methane concentrations peak in the anoxic core of the ETNP OMZ. We then used a combination of water and sediment incubations, along with stable isotope tracers and molecular analyses, to quantify sediment methane flux, methanogenic potential and pelagic methane oxidation potentials. We hypothesised that all sediments would contain active methanogens, but that their activity would be controlled by the oxygen concentration in the bottom-water. Further, the methane released from the sediments is then oxidised by aerobic and/or anaerobic methanotrophs in the water column as it moves towards the OMZ margins. To the best of our knowledge, this is the first study to combine biogeochemical with molecular microbial data, in order to better understand the origins and fate of methane in the ocean's largest OMZ.

## Materials and methods

### Sample sites

This study comprised two cruises in the ETNP: the first (D373, 11 December 2011–13 January 2012), which focused on the water column (0–4000 m), was structured around 6 ‘offshore' sites located along 92.5^o^W, between 8 and 13°N (Supplementary Figure S1). The second (JC097, 28 December 2013–10 February 2014), concentrated on the continental shelf and slope, 70–150 km off the Guatemalan coast (Supplementary Figure S1) and here, both sediments and water column samples were collected. A standard conductivity–temperature–depth rosette, comprising 24 Niskin (20 litre) bottles and a Sea-Bird 24 electronics system (fluorimeter, altimeter, photosynthetically active radiation and oxygen sensors, and so on) was used to collect water and a multi-corer (Mega Corer, OSIL, Havant, UK) was used to recover intact cores of sediment and overlying water.

### Water column gas and nutrient profiles

High-resolution (5–15 m) water column profiles (*n*=21) were constructed to define the OMZ and locate the methane. To minimise atmospheric contamination, water for methane analysis was discharged from the Niskin bottles into 12.5 ml gas-tight vials (Labco, Lampeter, UK) via Tygon tubing and allowed to overflow three times before capping, temperature equilibration and head-spacing (2 ml helium (BOC, Guildford, UK)). Methane was measured on-board using a gas chromatograph fitted with a flame ionisation detector (gas chromatography/flame ionization detector Agilent Technologies (Santa Clara, CA, USA), see Sanders *et al.* (2007) for details). Oxygen concentrations were measured by the Sea-Bird sensor (Bellevue, WA, USA) (with a limit of detection (LOD) ~1.4 μmol l^−1^) and nitrite was measured using a segmented flow auto-analyser (Skalar, Breda, Netherlands; LOD=0.05 μmol l^−1^, Nicholls *et al.*, 2007).

### Sediments as a methane source

Sediment-water flux was determined using intact cores and the methanogenic potential of discrete layers was quantified using slurries. As the conductivity–temperature–depth could not sample closer than ~10 m from the seabed, the water overlying the sediment (*n*=3 from the least disturbed core) was sampled, as above, to measure the methane concentration as close to the seabed as possible (<15 cm). Next, six sediment mini-cores were subsampled from three of the large cores (using Perspex tubes, 3.4 × 25 cm), sealed with rubber bungs and transferred to a temperature controlled (10 °C) tank. This was repeated at 16 locations ranging in seabed depth from 100 to 900 m.

Methane flux was quantified by measuring methane in the overlying water before and after a sealed 24-h incubation. First, the overlying water was degassed by bubbling (2 min) with oxygen-free nitrogen (BOC), to ensure all cores were incubated under the same hypoxic conditions (precise concentration verified using an oxygen micro-sensor, Unisense, Aarhus, Denmark) and that the majority of ambient methane was removed (previous experiment had demonstrated that 2 min was sufficient to remove >90% methane). Water samples were taken from each mini-core after degassing (T_0_), they were then sealed with bungs with inbuilt magnetic stirrers, and left for 24 h in the dark until a second water sample (T_final_) was taken for methane analysis. The daily flux of methane was calculated as the increase between T_0_ and T_final_.

To identify the sediment layer with the greatest methanogenic potential, additional large sediment cores (six locations, Table 1) were carefully extruded and ~4 ml of sediment and 3 ml of bottom water (overlying the cores) was transferred to gas-tight vials using a truncated 1 ml syringe (to minimise air contamination) to create a slurry. The headspace and water was purged with helium for 2 min to deoxygenate the vials and optimise conditions for methanogenesis. The methane concentration in the headspace was measured by gas chromatography/ flame ionization detector 4–8 times over the following 4–12 days and between measurements vials were kept at 12 °C in the dark. Following the first two experiments (550 m and 650 m), only the top 5 cm was used for further sites.

The concentration of sulphate, nitrite and nitrate in the sediment porewater was measured in eight large cores from four different locations (150, 350, 550 and 750 m seabed depth) by ion chromatography (Dionex, Sunnyvale, CA, USA; for sulphate) and segmented flow auto-analyser (Skalar for nitrite and nitrate), after separating the porewater from the sediment by centrifugation. Finally, hydrogen sulphide was measured in the cores by inserting a calibrated, miniaturised amperometric sensor (Unisense) into an extruded portion of the core, from the side, at 2 cm intervals.

### Aerobic and anaerobic water column methane oxidation

We set up four experiments using ^13^C-labelled methane to quantify the potential for aerobic and anaerobic methane oxidation in the water column (Supplementary Figure S2). First, we set up short time experiments with water from the upper margin of the OMZ, where oxygen is at the LOD (200 and 226 m). Seawater saturated with 99.9% ^13^C-CH_4_ was used to spike the samples with 3.3 nmol ^13^C-CH_4_ (264 nmol L^−1^) to avoid the need for a headspace and, thus, maintain ambient oxygen conditions. Samples were fixed (100 μl of 12.2 m HCl) at 3–5 time points over 10–15 days to track the accumulation of dissolved inorganic carbon (^13^C-DIC).

Second, we set up dose-response experiments (65 and 200 m), whereby we varied the injection volume to give a range of methane concentrations (44–790 nmol l^−1^) to assess the extent to which the methanotrophic community was substrate limited. These were left for the duration that the samples took to get back to the UK (5 months), without a headspace, before fixing.

Third, we started more widespread long-term incubations (eight locations) at one methane concentration (264 nmol l^−1^) and after fixing a sample within 2 min (control), we incubated the remaining samples for 5 months at 12 °C in the dark. Finally, to test for the potential for nitrite-dependent anaerobic methane oxidation (N-DAMO) we incubated water from five depths spanning the upper boundary and into the core of the OMZ, where nitrite and methane were both present (235–412 m, Supplementary Figure S2 and Table 2) with ^13^C-CH_4_ (3.4 μmol l^−1^) and ^15^N-NO_2_ (11.4 μmol l^−1^), just ^13^C-CH_4_ or no spike. These samples were taken from depths with no detectable oxygen but, to ensure complete anoxia in the water, we introduced a 2 ml helium headspace before adding the gas spikes. For all four types of incubations, we included a reference sample (no spike, fixed at same time as samples), a spiked-control (spiked with ^13^C-CH_4_ and/or ^15^NO_2_ and killed at the beginning of the experiment) and three technical replicates of each treatment. Microbial activity was stopped, and any resulting ^13^C-DIC converted to ^13^C-CO_2_ for analysis, by injecting HCl through the septa (as above). See Supplementary Figure S2 for sample location details.

Upon return to the UK, all unfixed samples were fixed and a 2 ml helium headspace was introduced into those incubated without one. To confirm the initial CH_4_ concentration, spiked-control samples were analysed on a gas chromatography/flame ionization detector and then the ^13^C-DIC (and ^15^N-N_2_ where necessary) was quantified using an elemental analyser interfaced with a continuous flow isotope ratio mass spectrometer (Sercon 20–22, Sercon Group, Crewe, UK), calibrated against sodium bicarbonate (0–4 mM for DIC) or air (for N_2_).

### Molecular analysis

Water (see Supplementary Table S1 for details) was filtered either through stand alone pumps or Nalgene filtration units (Supor, Pall, Port Washington, NY, USA; ø 293 mm for stand alone pumps or ø 47 mm for Nalgene units, 0.2 μm pore size filters). Filters were immediately frozen in liquid nitrogen, placed in –80 °C freezer and transferred to the UK for DNA extraction. Sediments (seabed depth 222 , 342, 550, 650 and 657 m) were collected from the top 2 cm of the sediment cores into 2 ml cryovials and frozen (as above) until DNA extraction. Details of the extraction process and downstream analysis are given in Supplementary Information.

### Accession numbers

The DNA sequences reported in this study were deposited in the EMBL database under the accession numbers LT575999–LT576028.

## Results

### Water column profiles

**Figure 1:**
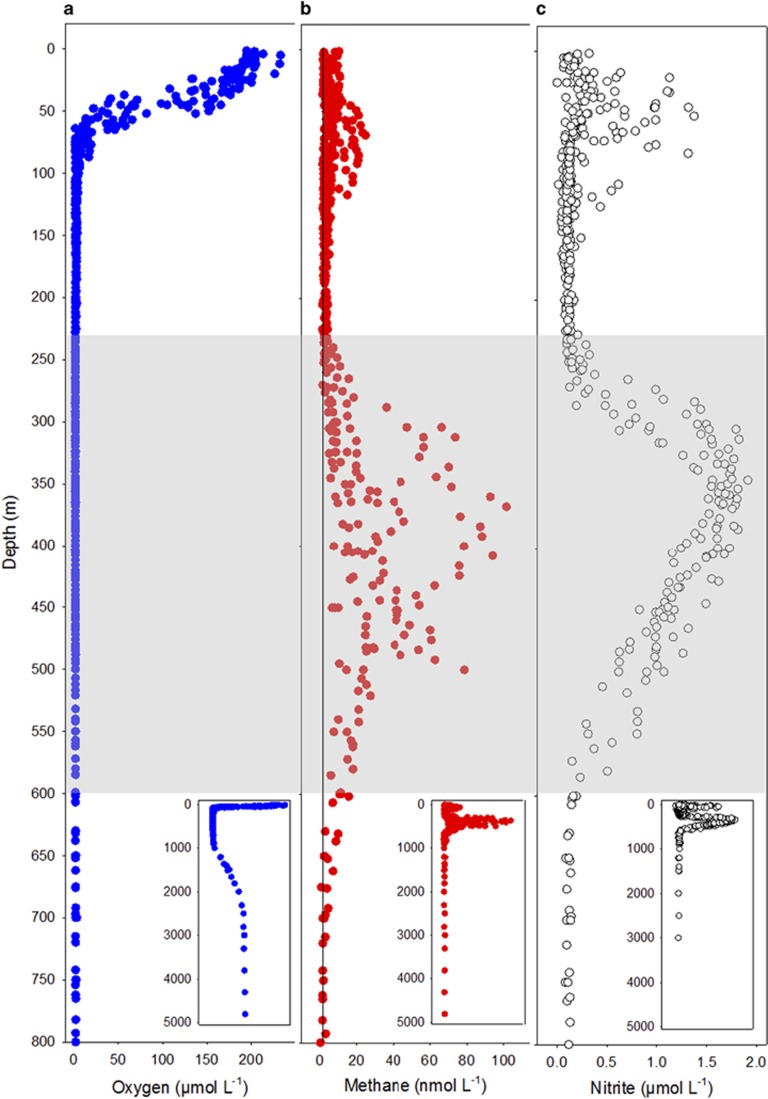
Water column profiles of (**a**) oxygen, (**b**) methane and (**c**) nitrite were constructed from conductivity–temperature–depth (CTD) deployments with the top 800 m shown in main panels and all data (0–5000 m) shown in inset plots. The shaded grey segment indicates the core of the OMZ (230–600 m) where oxygen is below LOD (~1.4 μmol l^−1^) and methane and nitrite are accumulating. Black line indicates atmospheric equilibration of methane at the depth-specific temperature and salinity.

Over the two cruises, we constructed 21 water column profiles covering seabed depths ranging from 55 to 5320 m. Oxygen declined steeply from an average of 193 μmol l^−1^ in the top 20 m, to 6.4 μmol l^−1^ at 80 m and then slowly until it went below our LOD (1.4 μmol l^−1^) at 230 m (Figure 1). Below 800 m, oxygen returned to detectable concentrations and reached 100 μmol l^−1^ (30% saturation) at 2500 m where it remained stable until the seabed (>5000 m). The baseline nitrite concentrations were 0.05 (LOD) to 0.2 μmol l^−1^ but within the OMZ, at 275–600 m, there was a large, secondary nitrite maximum of up to 1.8 μmol l^−1^ at 345 m and in the epipelagic waters, a smaller, primary maximum at around 50 m (max 1.37 μmol l^−1^) in most profiles (Figure 1). The true anoxic core of the OMZ was where oxygen was below the LOD and a clear secondary nitrite maximum was present (230–600 m).

Methane was supersaturated relative to the atmosphere throughout the water column and there was a clear peak around 250–600 m (Figure 1). The maximum concentration (102 nmol l^−1^) was measured at 368 m on the continental shelf where the seabed depth was 506 m, although very close to the sediments (<15 cm) 254 nmol l^−1^ was measured. Outside the core of the OMZ, the methane was consistently above atmospheric equilibration at 3–5 nmol l^−1^, except for a small epipelagic methane peak (Figure 1), which was only found in some of the profiles (maximum concentration 25 nmol l^−1^ at 65 m).

### Methane flux, methanogenesis and the methanogen community

**Figure 2:**
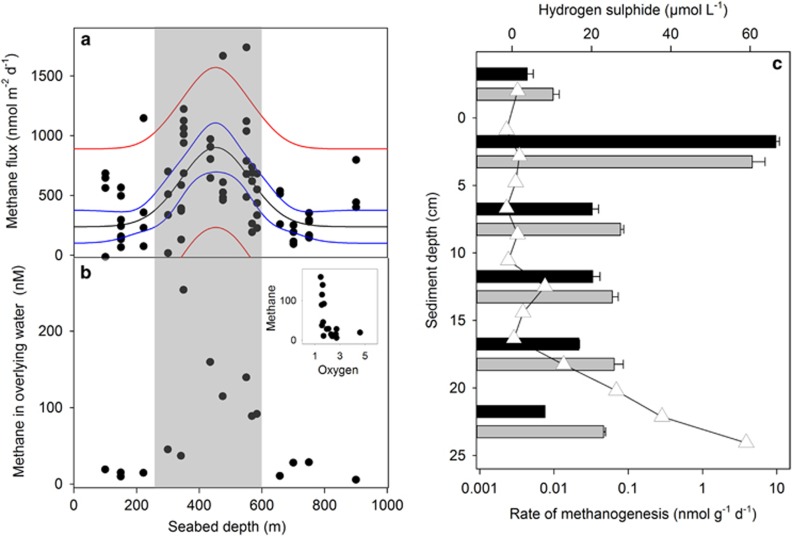
Spatial variation in (**a**) methane flux from sediment mini-cores and (**b**) methane concentration in the bottom, water measured above the cores (<15 cm from sediment surface), plotted against seabed depth. The shaded area indicates where the conductivity–temperature–depth (CTD) profiles measured anoxic bottom water (~10 m from seabed). The Gaussian peak, 4-paramter regression line (black), 95% confidence (blue) and prediction (red) intervals are shown in **a**. Inset, oxygen concentration and methane concentration in the bottom water for the points shown in **b**. (**c**) Depth profiles of methanogenic potential (displayed on a logarithmic scale) measured in slurries at five discrete sediment layers from 0 to 25 cm in cores from 550 m (grey) and 650 m (black). As a result of this strong depth decay, all further experiments focused on the top 5 cm only. Hydrogen sulphide profile for a core from 550 m (650 m data unavailable) overlain in unfilled triangles.

In the intact, anoxic core incubations, methane flux averaged 262±65.5 nmol m^−2^day^−1^, peaking at 1007 nmol m^−2^day^−1^ at 550 m and with the slowest of 162 nmol m^−2^day^−1^ measured at 300 m (Figure 2a). Although all sediment cores were degassed to remove oxygen (that is, optimal conditions for methanogenesis), methane efflux was greater in sediments from locations where the OMZ intersected the shelf (indicated by shaded area on Figure 2a) compared with those with oxygenated (2–4.6 μmol l^−1^) bottom-water (*X*^2^_(1)_=13.261, *P*<0.0001). The efflux of methane from the sediments was positively correlated with the concentration of methane in the bottom-water (*X*^2^_(1)_=23.233, *P*<0.0001), which ranged from 6 to 254 nmol l^−1^ (Figure 2b). Further, there was a strong, non-linear inverse relationship between the concentration of methane and oxygen in the bottom-water (Figure 2b, inset, LOD~1.4 μM O_2_).

Incubating anoxic sediment slurries from discrete depth intervals from two locations revealed that the bulk of the methanogenic potential was in the surface sediments (Figure 2c) and so all further experiments were focused on this layer (Table 1). Methanogenesis was also detected in the overlying water (0–5 cm above sediment) and in sediments down to 25 cm, but in the uppermost layer the rate was at least an order of magnitude higher than any other depth (Figure 2c). The greatest potential was measured in sediment from 750 m (88 nmol g^−1 ^day^−1^, Table 1). Hydrogen sulphide was detected in two out of the six cores in which it was measured; at 550 m the concentration reached 59 μmol l^−1^ at 25 cm (Figure 2c) and in a core from 350 m it reached 219 μmol l^−1^ at 23 cm. The porewater profiles (Supplementary Figure S3) revealed that in the top 2 cm of sediment, where methane production was most active, sulphate (23 mmol l^−1^), nitrite (2.8 μmol l^−1^) and nitrate (86 μmol l^−1^) were similar to, or above, bottom-water concentrations.

**Figure 3:**
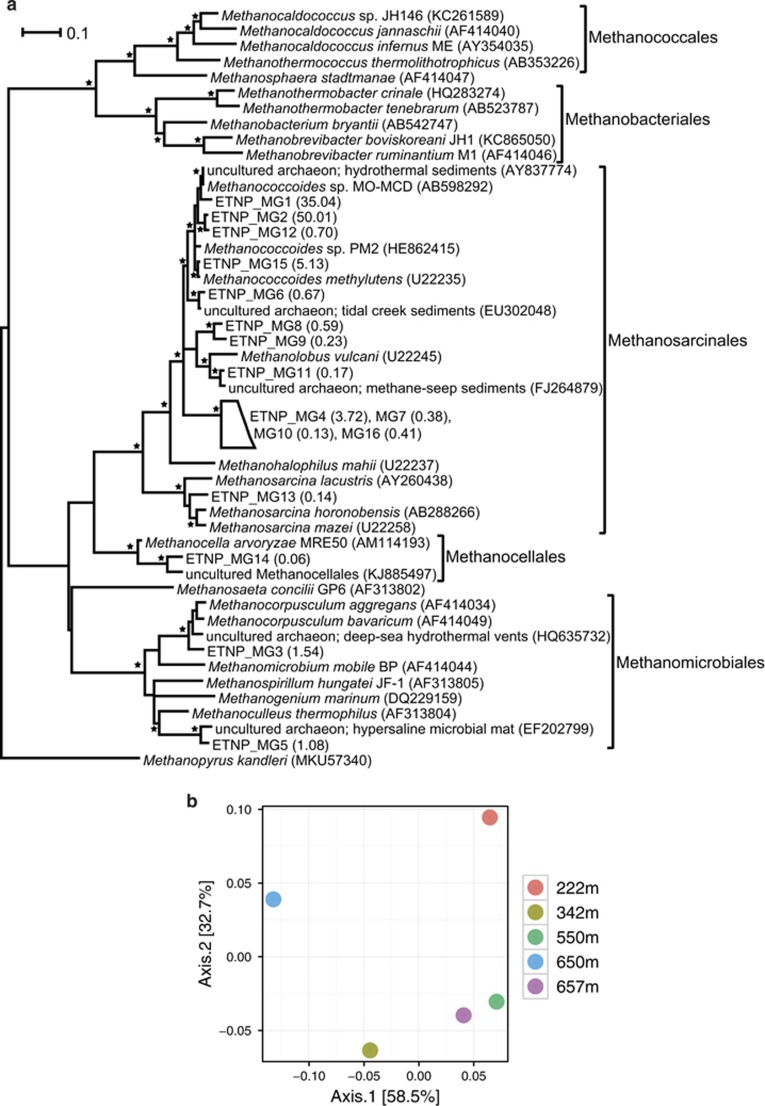
Methanogen community at five points across the seabed sediment. (**a**) Maximum likelihood tree of representative partial *mcrA* sequences (about 362 bp). Collapsed branches are indicated by a polygon. The *mcrA* gene of *Methanopyrus kandleri* (MKU57340) was used as the outgroup. Asterisks indicate local support values over 75%. The number in parenthesis following the ETNP_MG sequences indicates the % relative abundance of each OTU over the total number of OTUs (see Supplementary Table S2 for details). The bar represents 0.1 average nucleotide substitutions per base. (**b**) Principal coordinate analysis (PCoA) plot of the methanogen community based on the *mcrA* sequences and a maximum likelihood tree, and, constructed with the weighted Unifrac metric.

A total of 126 303 *mcrA* gene sequences from the top 2 cm were retrieved and clustered into 16 operational taxonomic units (OTUs), hereafter named as ETNP_MG. The majority of sequences (50% of sequences represented by ETNP_MG2 and 35% represented by ETNP_MG1) were 96–97% similar to *Methanococcoides* sp. (Supplementary Table S2). Most of the OTUs (13 out of 16) clustered within the order Methanosarcinales, whereas two OTUs (ETNP_MG3 and ETNP_MG5) clustered within the order Methanomicrobiales and one (ETNP_MG14) within the order Methanocellales (Figure 3a). The *Methanococcoides*-like species dominated all five seabed samples, which displayed a similar level of intra-sample diversity (assessed by Shannon and Simpson indices, Supplementary Table S2). Principal coordinate analysis indicates that most of the variation (91.2%) in the methanogen community is explained by the first two principal coordinates (58.5% of the variation explained by axis 1 and 32.7% by axis 2, Figure 3b). The most separated communities, by the first coordinate, are those from 650 to 222 m, because of unique Methanomicrobiales (ETNP_MG5) and Methanocellales (ETNP_MG14) sequences at 650 m and Methanosarcinales at 222 m. The community at 342 m is also somewhat separated from the others, as it is the only one with ETNP_MG3 Methanomicrobiales-like sequences.

### Methane oxidation and methanotrophs

**Figure 4:**
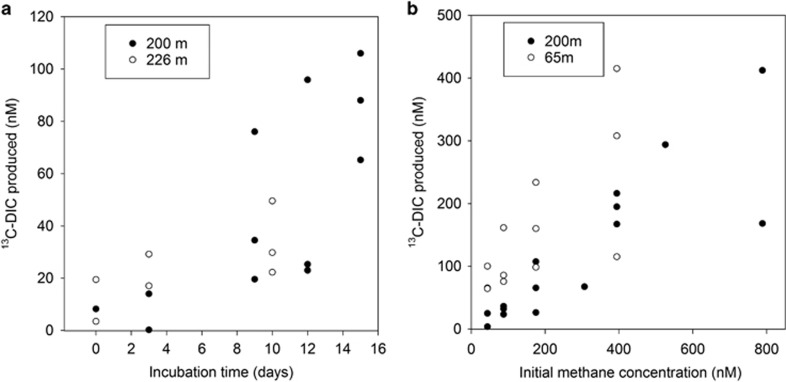
Methane oxidation measured as ^13^C-DIC accumulation, (**a**) over a 13-day time series with water from 200 to 226 m, on the upper margin of the OMZ, where oxygen is close to LOD (~1.4 μmol l^−1^) and methane is rising above background concentrations, and (**b**) over 5 months with varying initial concentrations of methane.

Methane oxidation was measured, through the accumulation of ^13^C-DIC, in short-term (10–15 days) time series incubations with water from the uppermost margins of the OMZ (Figure 4a and Supplementary Figure S2); ^13^C-DIC was produced at a rate of 3.0–5.9 nmol l^−1^ day^−1^, and, after 15 days, 26% of the ^13^C-CH_4_ had been oxidised to ^13^C-DIC. In the epipelagic zone, we could not measure any methane oxidation at 47 m but we did at 65 m (Table 2). Our dose-response experiments indicate that the methanotrophs can oxidise methane at concentrations much higher than the ambient concentration (Figure 4b). Water from both the epipelagic (65 m) and mesopelagic waters (200 m, where oxygen was below detection) oxidised increasing amounts of ^13^C-CH_4_ to ^13^DIC with increasing initial methane spike (Figure 4b) and there was a good correlation between methane oxidised and ^13^C-DIC produced (R^2^=0.96). This relationship between ^13^C-DIC produced and starting methane concentration was linear (*R*^2^_(200m)_=0.94 and *R*^2^_(65m)_=0.54) for the range of concentrations tested (85–760 nmol l^−1^) and the slope (b^1^_(200m)_=0.0055 and b^1^_(65m)_=0.0057) of the relationship was similar for the two different water samples (Figure 4b).

Long-term (5 months) incubations from eight different locations (47–228 m), yielded mixed results, with methane oxidation being undetectable in some vials (Table 2). The greatest amount of ^13^C-DIC produced was at 200 m where, following a 3.3 nmol spike of ^13^C-CH_4_, 0.6 nmol ^13^C-DIC was measured in the water after 5 months. In the shorter incubations, water from the same location, produced a similar amount in only 10 days (Figure 4a), which indicates methane oxidation did not continue linearly during the 5 months. For comparison, if a total of 0.6 nmol ^13^C-DIC in the vial accumulated linearly, after 150 days, it would equate to 0.42 nmol l^−1^ day^−1^, which is 14 times slower than that measured in the short-term incubations.

Water incubated with ^13^CH_4_ and ^15^NO_2_^−^ did produce both ^13^DIC (0.9–15.4 nmol) and ^29+30^N_2_ (8.9–80.5 nmol), and the relative proportions produced varied across depths with high rates of ^29+30^N_2_ production at 235 and 264 m, indicative of nitrite reduction alongside methanotrophy (Table 2 and Supplementary Figure S2).

**Figure 5:**
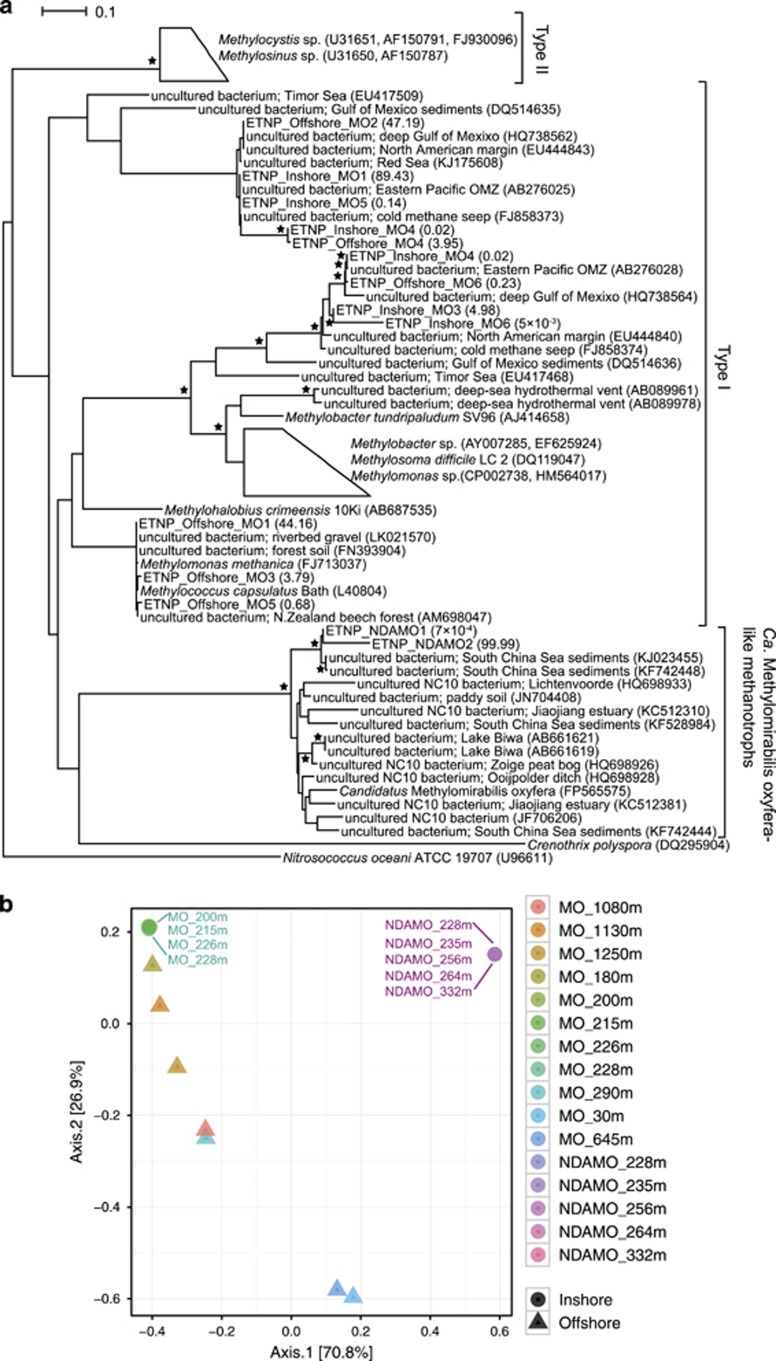
Aerobic and anaerobic methanotroph community of the water column. (**a**) Maximum likelihood tree of representative partial *pmoA* sequences (about 347 bp). Collapsed branches are indicated by a polygon. The *amoA* gene of *Nitrosococcus oceani* ATCC 19707 (U96611) was used as the outgroup. Asterisks indicate local support values over 75%. The number in parenthesis following the ETNP_Inshore_MO, ETNP_Offshore_MO and ETNP_NDAMO sequences indicates the % relative abundance of each OTU over the total number of Inshore, Offshore and N-DAMO OTUs, respectively (see Supplementary Table S3 for details). The bar represents 0.1 average nucleotide substitutions per base. (**b**) Principal coordinate analysis (PCoA) plot of the methanotroph community based on the *pmoA* sequences and a maximum likelihood tree, and, constructed with the weighted Unifrac metric. Circles indicate inshore samples and triangles indicate offshore samples. MO stands for aerobic methanotrophs and N-DAMO for anaerobic methanotrophs. MO_Inshore and N-DAMO communities are shown by overlapping green and purple circles, respectively.

Methanotrophic bacteria were targeted in waters offshore (30–1250 m depth) and closer to the coast (200 and 228 m). Analysed aerobic *pmoA* sequences in the offshore samples (6202 in total) were clustered into six OTUs, hereafter called ETNP_Offshore_MO, and in the inshore samples (363 816 in total) were clustered into six OTUs, hereafter called ETNP_Inshore_MO. The sequences from both the offshore and inshore samples were highly similar (97–100% BLAST similarity) to uncultured bacteria from marine environments (Supplementary Table S3, Figure 5a). The vast majority of the offshore sequences are represented by two OTUs, that is, ETNP_Offshore_MO1 (44.16% of sequences) and ETNP_Offshore_MO2 (47.19% of sequences). Similarly, ETNP_Inshore_MO1 represents the majority (89.43%) of the analysed inshore sequences. Phylogenetic analysis shows that all the OTUs cluster within known type I methanotrophs (Figure 5a). Among them, three OTUs of the offshore samples (ETNP_Offshore_MO1/MO3/MO5) sit within a sub-cluster of the family Methylococcaceae including *Methylococcus* and *Methylomonas* species.

The diversity (based on Shannon and Simpson indices) within all the analysed samples and particularly of the inshore ones is small, with the most diverse sample being that of 290 m offshore (Shannon=1.19, Simpson=0.64; Supplementary Table S3). The principal coordinate analysis plot also shows a very close proximity of all the inshore samples (that is, green circles on Figure 5b practically overlap), whereas there is some variance among the offshore samples, as indicated by their good separation along the second principal coordinate, that is, axis 2, explaining 26.9% of the observed variance (triangles in Figure 5b).

However, most of the principal coordinate analysis variance is explained by the first principal coordinate (axis 1, explaining 70.8% of the variance), which is mainly driven by the divergence of the anaerobic methanotroph community (overlapping purple circles on Figure 5b) and, to a lesser extent, by the divergence of two offshore aerobic methanotroph samples (30 and 645 m; blue triangles on Figure 5b). The diversity within the two OTUs of the anaerobic methanotrophs (ETNP_NDAMO1 and ETNP_NDAMO2) is minimal (see Shannon and Simpson indices, Supplementary Table S3). Indeed, phylogenetic analysis placed both of these OTUs into a separate and well-defined cluster, related to the *Candidatus* Methylomirabilis oxyfera anaerobic methanotroph (Figure 5a).

## Discussion

Here we have shown that biological methanogenesis, in the surface layer of the seabed sediments, is a major source of methane to the ETNP OMZ. These are the first direct measurements of methane production in sediments from this region. The reactivity of these sulphate and nitrate-rich surface sediments highlights the potential importance of non-competitive methanogenesis to the marine methane pool. Our oxygen profiles agree with previously published data for the ETNP OMZ (Burke *et al.*, 1983; Sansone *et al.*, 2001, 2004; Pack *et al.*, 2015) and show oxygen to be ⩽1.4 μmol l^−1^ between 200 and 800 m. However, as a secondary nitrite maximum occurs when oxygen is below 0.05 μmol l^−1^ (Thamdrup *et al.*, 2012), we used this profile to define the true core of the OMZ (230–600 m). In addition, we have presented evidence for microbial methane oxidation, which can be sustained under a wide range of oxygen (<1.4–65 μmol l^−1^) and methane (44–790 nmol l^−1^) concentrations, potentially controlling the release of methane emissions from the OMZ.

The highest potential for methanogenesis is in the top 2 cm of seabed, in the presence of ample sulphate, nitrate and nitrite (as alternative electron acceptors) and >20 cm above the hydrogen sulphide peak (Figure 2c, Table 1). The co-occurrence of the greatest potential for methanogenesis and highest concentration of sulphate indicates that this is likely to be non-competitive methanogenesis and this is supported by the methanogen community findings. The majority of methanogens in all the analysed samples (97.31% of total sequences) clustered within the family Methanosarcinaceae and the dominant OTUs were similar to *Methanococcoides* sp. deriving from sub-seafloor sediments (Imachi *et al.*, 2011) or estuarine mudflats (Watkins *et al.*, 2012). *Methanococcoides* sp. have often been isolated from marine sediments (for example, Singh *et al.*, 2005; Lazar *et al.*, 2011; Webster *et al.*, 2015) and they are obligatory methylotrophic methanogens, that is, utilising only non-competitive substrates, such as methanol or methylamines (Garcia *et al.*, 2000; Ferry, 2010).

Although we could not find other direct measurements of methanogenesis in the ETNP OMZ, there are data reported from other locations. Krüger *et al.* (2005) reported rates of methanogenesis in sediment surface slurries from eight different marine sites in the Atlantic, Pacific and Arctic Oceans and the North and Baltic Seas, and their results (0.01–0.1 μmol g^−1^ day^−1^) agree well with our measurements using the same technique (0.001–0.09 μmol g^−1^ day^−1^, Table 1). They noted that the highest methanogenic potentials were measured in regions with high input of organic matter from the water column (Krüger *et al.*, 2005). The only intact core experiment (Crill and Martens, 1983) to report marine methane flux was performed on coastal sediments from Cape Lookout Bight, and showed a similar range (0.18–1.56 μmol m^−2^ day^−1^) to those found in our ETNP sediments (0.16–1.01 μmol ^ ^m^−2^ day^−1^).

The potential for methanogenesis was markedly reduced below the top 2 cm and we propose that this is linked to organic carbon supply raining down from above, which the surface methanogens can preferentially access. Continental shelves are known for high productivity and therefore, the delivery of carbon to the seafloor is high relative to less productive areas of the ocean (Ramaswamy *et al.*, 2008; Fennel, 2010).

We propose that this benthic methanogenesis supplies the water column with methane, which persists far offshore. The location of the methane peak (250–700 m), agrees well with other ETNP studies but the magnitude in our study (102 nmol l^−1^ using the conductivity–temperature–depth and 254 nmol l^−1^ very close to the sediment surface using Mega-Cores) was considerably higher than previously reported (maximum 5–80 nmol l^−1^, Burke *et al.*, 1983; Sansone *et al.*, 2001, 2004 and Pack *et al.*, 2015). Variation in maximum concentrations found across the ETNP is likely due to proximity to the source of methane, dilution and slow microbial oxidation. The flux was greatest when oxygen concentration in the bottom-water was below the LOD (Figure 2a) and a clear plume, originating in the continental shelf slope and extending offshore, can be seen in our profiles (Supplementary Figure S1), both of which support our theory. Even when there was a wedge of oxygenated water between the OMZ and the seafloor, methane was supersaturated in the OMZ and the maximum concentration of methane decreased with distance offshore. Indeed, methane was only found to be over 35 nmol l^−1^ when the maximum water depth was between 350 and 650 m, and in the deeper water (seabed >750 m) the methane did not exceed 25 nmol l^−1^ even when oxygen and nitrite indicated true OMZ conditions. The close agreement between our potential methanogenesis rates and the flux data show that benthic methanogenesis could be responsible for all the methane measured in the bottom-water without the need to invoke additional methane sources, for example, seeps or dissociation of hydrates. Further, to the best of our knowledge, there are no reports of methane seeps in this OMZ.

The spatial alignment of the methane and nitrite peaks suggests that methane could be oxidised, in the presence of nitrite and the absence of measureable oxygen, that is, anaerobically. Our attempts to measure the potential for N-DAMO were inconclusive, and others (Padilla *et al.*, 2016) using a similar dual-isotope incubation technique, recently tried and failed to fully quantify this process in the ETNP OMZ. However, in our experiments, water incubated with ^13^C-CH_4_ and ^15^N-NO_2_ did produce ^13^C-DIC and ^29+30^N-N_2_ but the stoichiometry (Table 2) was not indicative of pure N-DAMO (3CH_4_ and 8NO_2_ produce 3CO_2_ and 4N_2_, Ettwig *et al.*, 2010), nor were the rates of ^13^C-DIC production consistently stimulated by addition of nitrite. Nonetheless, sequences from N-DAMO-like bacteria were detected in all the targeted water depths. The sequences belonged to just two closely related phylotypes (ETNP_NDAMO_1 and ETNP_NDAMO_2; Figure 5,Supplementary Table S3) affiliated with uncultured anaerobic methanotrophs from South China Sea sediments (Chen *et al.*, 2014, 2015). They clustered within the *Candidatus* Methylomirabilis oxyfera lineage, which is known to couple anaerobic methane oxidation to the reduction of nitrite (Ettwig *et al.*, 2010; Haroon *et al.*, 2013) and, although they are well described in lakes (Deutzmann and Schink, 2011; Kojima *et al.*, 2012), paddy soils (Wang *et al.*, 2012) and peatlands (Zhu *et al.*, 2012), the ecological role of these phylotypes in marine environments has only recently been addressed (Chen *et al.*, 2014; Li-Dong *et al.*, 2014). More recently, Padilla *et al.* (2016) reported transcriptionally active Methylomirabilis-like NC10 phylotypes in all their ETNP sites, off the North Mexican coast, with the abundance of 16S rRNA transcripts peaking in the core of the OMZ, thereby confirming marine OMZs as a niche for such phylotypes. In agreement with recent findings in the South China Sea (Chen *et al.*, 2015), we show that these marine phylotypes form a separate cluster from their equivalent freshwater phylotypes.

We were able to confirm the potential for aerobic methane oxidation in the OMZ of ETNP by measuring the conversion of ^13^C-CH_4_ to ^13^C-DIC over relatively short timescales (<2 weeks). We artificially raised the methane concentration, to ensure that ^13^C-CH_4_ (rather than ^12^C-CH_4_) constituted the overwhelming majority of the methane available for oxidation. However, we can use our dose-response experiment to approximate ambient rates of methane oxidation. For example, at 200 m the average methane concentration was 3.3 nmol l^−1^ so although we measured 4.5 nmol l^−1^ day^−1^ (incubated with 300 nmol l^−1^ CH_4_) *in situ* we would expect 0.0495 nmol l^−1^ day^−1^ with a turnover time of 67 days. Even in anoxic incubations (LOD for oxygen), ^13^C-DIC was produced following a spike of ^13^C-CH_4_ and so it is reasonable to predict that methanotrophs are oxidising methane right at the margin of the OMZ core and our measurements fall within the range recently reported for methane oxidation in the ETNP (0.000034–4 nmol l^−1^ day^−1^, Pack *et al.*, 2015).

Molecular analysis confirmed the presence of aerobic methane oxidisers at a wide range of depths (ranging from 30 to 1250 m) in both offshore (ETNP_Offsshore_MO) and coastal (ETNP_Inshore_MO) waters. The majority of methanotrophs from inshore waters (99.96% of sequences) were phylogenetically related (>97% similarity, Figure 5,Supplementary Table S3) to uncultured bacteria detected in the ETNP (Hayashi *et al.*, 2007). The methanotrophs in the offshore samples were somewhat more diverse, with some similar to those in the inshore samples and others forming a separate sub-cluster with known Methylococcaceae species (for example, *Methylomonas methanica* and *Methylococcus capsulatus* str. Bath, Figure 5). This could be partly attributed to the difference in the range of depths from which the samples were obtained, that is, offshore samples were collected from depths between 30 and 1250 m, whereas inshore samples were collected from a much narrower range of depths (200–228 m). Depth-related differences in the aerobic methanotroph community along vertical water horizons have been reported elsewhere (for example, Tavormina *et al.*, 2010, 2013). Such differences may be related to the physical transport of waters, harbouring distinct microbial communities, which, along with environmental selection and spatial separation, has been shown to shape the distribution of marine microbes (Wilkins *et al.*, 2013; Steinle *et al.*, 2015). The diversity of methanotroph phylotypes in the water column is likely controlled by environmental factors rather than geographical proximity, and the same phylotypes may be adapted to a range of methane concentrations (Tavormina *et al.*, 2008). Indeed, a later study of Cu-MMO phylotypes from the Costa-Rican OMZ showed that methane concentration did not predict the occurrence, abundance or distribution of any phylotypes; instead environmental factors such as depth, salinity, temperature and dissolved oxygen concentrations accounted for most of the observed phylotype variance (Tavormina *et al.*, 2013). Aerobic methanotrophs in both offshore and inshore samples clustered within type I, whereas no sequences were affiliated to type II methanotrophs, which is in accordance with the findings in other marine environments (Schubert *et al.*, 2006; Tavormina *et al.*, 2008; Wasmund *et al.*, 2009; Schmale *et al.*, 2012). The lack of close affiliation of marine phylotypes with established methanotroph lineages has been reported previously and it has been linked to specialisation of these phylotypes in marine environments and to the rather small representation of marine methanotroph sequences in public databases (Tavormina *et al.*, 2008; Wasmund *et al.*, 2009).

Here we present clear evidence for microbial methanogenesis in the continental shelf sediments fuelling the ETNP OMZ methane plume, which is sustained several 100 km offshore, despite biological oxidation. Molecular analyses support the methanogenesis and methanotrophy potentials presented, however, more studies are needed to fully unravel the diversity of pelagic methanotrophs and to determine the precise electron acceptors for anaerobic methane oxidation.

## Figures and Tables

**Table 1:** Methanogenic slurry potentials in which the methane concentration was measured daily

*Seafloor depth (m)*	*Mean sediment depth (cm)*	*Methanogenesis (nmol g*^−*1*^ *day*^−*1*^)	*s.e.*	*Number of replicates*
150	Water	0	0.003	4
	1	0.67	0.229	6
	3	0.44	0.402	6
350	Water	0	0.001	2
	1	17.87	7.82	4
	3	2.19	1.85	4
550	Water	0.01	0.002	4
	2.5	4.66	2.288	2
	7.5	0.08	0.008	2
	12.5	0.06	0.012	2
	17.5	0.06	0.022	2
	22.5	0.05	0.003	2
550	Water	0.01	0.012	4
	1	13.09	5.213	6
	3	0.27	0.028	6
650	Water	0.004	0.0008	8
	2.5	9.73	1.162	4
	7.5	0.03	0.007	4
	12.5	0.03	0.008	4
	17.5	0.02	0.001	2
	22.5	0	-	1
750	Water	0.002	0.0025	2
	1	87.84	15.32	4
	3	0.32	0.040	4

Mean rates of methane production (± s.e.) are presented for discrete sediment depth intervals and for the water immediately overlying the sediment. The rate of methanogenesis was calculated over 3–5 days depending on the linearity of production with time. Cores were collected from six locations with varying seabed depths. Two separate locations, where the seabed depth was 550 m, were targeted.

**Table 2:** Methane oxidation in long-term water incubations at 12 °C

*Latitude/longitude*	*Sample water depth (m)*	*Ambient oxygen (μmol l*^−*1*^)	*Ambient methane (nmol l*^−*1*^)	^*13*^*C-DIC produced (nmol)*	*Initial isotope spikes*	^*15*^*N-N*_*2*_ *produced (nmol)*
13°21 N/91°23 W	195	1.9	3.1	0.2±0.03	264 nM ^13^CH_4_	NA
	200	1.5	2.8	0.6±0.34		
	205	⩽1.4	4.4	0.1±0.09		
	210	⩽1.4	3.4	0.20±0.22		
13°15 N/91°08 W	47	132.1	4.8	−0.1±0.06		
	226	⩽1.4	2.1	−0.02±0.08		
13°16 N/ 91°08 W	65	11.4	2.4	0.2±0.02		
	228	⩽1.4	3.9	0.3±0.11		
13°25 N/ 91°23 W	235	⩽1.4	4.3	2.6±1.25^a^		
	235*			2.4±1.11^a^		80.5±30.11
13°16 N/ 91°08 W	322	⩽1.4	14.8	10.4±0.18^a^	3.4 μM ^13^CH_4_ and for *samples also 11.4 μM ^15^NO_2_^−^	
	322*			11.8±3.50^a^		16.0±2.64
	412	⩽1.4	33.9	12.3±4.03^a^		
	412*			15.4±0.78^a^		14.5±4.11
13°16 N/91°08 W	264	⩽1.4	9.4	1.3±1.20^a^		
	264*			12.0±6.00^a^		29.3±18.22
	256	⩽1.4	6.1	11.2±0.00^a^		
	256*			0.9±1.37^a^		8.9±0.61

Abbreviations: NA, not applicable; OFN, oxygen-free nitrogen.

Mean values (± s.e., *n*=3). Samples marked with '^a^' had a 2 ml OFN headspace throughout the incubation. Samples marked with * are those where ^15^N-NO_2_ (11.4 μmol l^−1^) was introduced and the production of ^15^N-N_2_ measured after 5 months. Where oxygen was at or below the limit of detection it was assumed to be ⩽1.4 μmol l^−1^.
