# Supplementary material for: Origin and fate of methane in the Eastern Tropical North Pacific oxygen minimum zone
Source: ISME J. 2017 Feb 28;11(6):1386–99. doi: 10.1038/ismej.2017.6 (PMC5437358; doi:10.1038/ismej.2017.6)
Supplement: Supplementary Methods and Tables [file ismej20176x1.docx]

**Origin and fate of methane in the Eastern Tropical North Pacific Oxygen Minimum Zone**

Panagiota-Myrsini Chronopoulou^1^*, Felicity Shelley^1^*, William J. Pritchard^1,2^, Susanna T. Maanoja^1,3^ and Mark Trimmer^1^

^1^School of Biological and Chemical Sciences, Queen Mary University of London, London, E1 4NS, UK

Current addresses:

^2^ Faculty of Life Sciences, University of Manchester, A4051 Michael Smith Building, Dover Street, Manchester, M13 9PT, UK

^3^ Department of Chemistry and Bioengineering, Tampere University of Technology, PO Box 541, FI-33101, Tampere, Finland

*These authors contributed equally to this work

**Supplementary Information**

**Molecular Analysis**

Seawater from the same location as that used to measure the potential for methane oxidation was filtered to collect material for molecular analysis. From some locations, water was discharged directly from the Niskins into 1L clear glass moulded infusion vials (Laboratory Precision Limited) and allowed to over flow for 2min. The water was then filtered through Supor®, ø 47mm, 0.2*μ*m pore size filters, using Nalgene filtration units and applying a gentle vacuum, and filters were placed in 2mL cryovials. For all other locations, stand-alone pumps (SAPs) were deployed at the targeted depth, left to filter water through Supor® or polycarbonate ø 293mm, 0.2*μ*m pore size filters and retrieved back on-deck. In both cases, filters were frozen immediately in liquid nitrogen and stored at -80°C until DNA extraction. Details of the water collection locations, filter types and volumes of water filtered are shown in Table S1. Sediment samples to target methanogenic potential were obtained from the top 2cm of sediment cores (following Mega Corer deployments), from seabed depth of 222m, 342m, 550m, 650m and 657m. Sediment was placed in 2mL cryovials (scooping sediment with a spatula from the top of the cores), immediately frozen in liquid nitrogen and stored in in -80°C until DNA extraction.

For the extraction of DNA from filters half of the ø 47mm or the equivalent size of the ø 293mm filter was placed in 2mL sterile tubes with zirconia/silica beads, 0.1mm (Stratech Scientific Limited). In the case of seabed sediments, 0.5-0.6g of sediment was used for the extraction. Potassium phosphate buffer (pH 8.0; 500*μ*L; 0.1M), and phenol-chloroform-isoamyl alcohol (25:24:1 v/v) (500*μ*L) were added to the extraction tubes. Cells were lysed by bead beating using a TissueLyser LT (Qiagen), at 50Hz for 1min. The rest of the extraction process was as described by (McKew *et al.*, 2007). DNA was allowed to air dry, and re-suspended in 55-65*µ*L of sterile ultra pure water.

Amplification of the functional genes was performed in a Bio-Rad MJ Research PTC-220 DYAD Thermal Cycler. The *pmoA* gene of aerobic methanotrophs was amplified with the primers A189/A682 (Holmes *et al.*, 1995). The 50*μ*L reaction mixture contained 1*μ*L of DNA, 0.4mM of each primer, 200*μ*M deoxynucleoside triphosphates, 20ng *μ*L^-1^ BSA, 1.25U of *Taq* DNA polymerase and 5mL of the reaction buffer supplied with the enzyme (New England Biolabs). PCR conditions were as follows: 94°C for 5min, 35 cycles of 94°C for 1min, 50°C for 1.5min and 72°C for 1min, with a final elongation of 72°C for 10min. The *pmoA* gene of anaerobic methanotrophs was amplified with the primer set A189_b/cmo682 (Luesken *et al.*, 2011) and PCR conditions as described in (Luesken *et al.*, 2011). The reaction mixture was as described above, except two times diluted DNA was used as template. For the *mcrA* gene of methanogens, the primer set ME/ME2 (Hales *et al.*, 1996) and conditions as described in (Hales *et al.*, 1996) were used. Reaction mixture was as above, except 2*μ*L of 10 times diluted DNA was used as template. PCR products were purified, using the QIAquick PCR Purification Kit (Qiagen) according to the manufacturer’s protocol, and they were used as template for next generation sequencing with internal primers of the amplified gene regions of *pmoA* or *mcrA* genes. The *pmoA* gene of aerobic methanotrophs was targeted using the A189 as forward primer in conjunction with the mb661 (internal of A682) (Costello & Lidstrom, 1999), the *pmoA* of anaerobic methanotrophs with the primers cmo182/cmo568 (Luesken *et al.*, 2011) and the *mcrA* gene with the primers mlas/mcrA-rev (Steinberg & Regan, 2008). Sequencing was performed on the Roche 454 FLX/FLX+ platform for the *pmoA* gene of offshore-deriving samples and on the Illumina MiSeq platform for the *pmoA* and *mcrA* gene of inshore deriving samples, at the Research and Testing Laboratory (<http://www.researchandtesting.com/index.php>).

**Processing of sequences and phylogenetic analysis**

The forward and reverse MiSeq reads were merged at the Research and Testing Laboratory using the Pear Illumina merger (Zhang *et al.*, 2014). All the downstream analysis of the paired-end MiSeq reads and of the 454 reads was performed in the QIIME pipeline and its associated modules (Caporaso *et al.*, 2010). Initially, all sequences were checked for the presence of correct MiSeq adaptors, 8bp barcodes (unique for each sample), and those containing errors in these regions were removed. Sequences <200bp, those with low quality scores (<25), and sequences containing homopolymers (>6bp) were also removed. All reads that passed the above mentioned quality controls were clustered into operational taxonomic units (OTUs), here referred to as ETNP_Inshore_MO or ETNP_Offshore_MO for ETNP-aerobic methane oxidation from inshore and offshore sampling stations, respectively, ETNP_NDAMO for ETNP-nitrite-dependent anaerobic methane oxidation and ETNP_MG for ETNP-methanogenesis related taxonomic units, using the USEARCH algorithm (Edgar, 2010). Clustering for *pmoA* sequences was done at the 90% similarity level (given the 3.5-times-higher nucleotide substitution rate of the *pmoA* compared to the 16S rRNA gene (Pester *et al.*, 2004) and for *mcrA* at 84% similarity (corresponding to 98% 16S similarity according to the polynomial fitting model of *mcrA* and 16S rRNA genes (Yang *et al.*, 2014)). The de novo chimera checker UCHIME was used to detect and remove all chimeric sequences (Edgar *et al.*, 2011). Representative sequences from each OTU were assigned taxonomy using BLAST (Altschul *et al.*, 1990), against the National Center for Biotechnology Information (NCBI) database. Sequences with no BLAST hit were excluded from downstream phylogenetic analysis.

Principal Coordinate Analysis (PCoA) was performed using the Unifrac distance metric (Lozupone *et al.*, 2006), as this is incorporated in the phyoseq R package (McMurdie & Holmes, 2013), after random subsampling so that all samples contained the same number of sequences (i.e. normalising to the sample with the minimum number of sequences). All representative sequences and their closest relatives from the BLAST analysis were aligned using the muscle algorithm (Edgar, 2004) and edited in MEGA6 (Tamura *et al.*, 2013). Maximum likelihood phylogenetic trees were constructed using MEGA6, after performing a “best model” analysis to select the best substitution model for each set of sequences (General Time Reversible model with discrete Gamma distribution rates among sites for both *pmoA* and *mcrA* sequences) according to BIC (Bayesian Information Criterion) (Hall, 2013). The trees were edited in Dendroscope (Huson *et al.*, 2007) and Adobe Illustrator.

Diversity indices (Shannon and Simpson indices, shown in Tables S2 and S3) were calculated using the vegan package version 2.2-1 in R (Oksanen *et al*., 2015).

**References**

Altschul SF, Gish W, Miller W, Myers EW, Lipman DJ. (1990). Basic local alignment search tool. *J Mol Biol* **215**:403–410.

Caporaso JG, Kuczynski J, Stombaugh J, Bittinger K, Bushman FD, Costello EK, *et al.* (2010). QIIME allows analysis of high-throughput community sequencing data. *Nat Methods* **7**:335–6.

Costello AM, Lidstrom ME. (1999). Molecular characterization of functional and phylogenetic genes from natural populations of methanotrophs in lake sediments. *Appl Environ Microbiol* **65**:5066–74.

Edgar RC. (2004). MUSCLE: a multiple sequence alignment method with reduced time and space complexity. *BMC Bioinformatics* **5**:113.

Edgar RC. (2010). Search and clustering orders of magnitude faster than BLAST. *Bioinformatics* **26**:2460–2461.

Edgar RC, Haas BJ, Clemente JC, Quince C, Knight R. (2011). UCHIME improves sensitivity and speed of chimera detection. *Bioinformatics* **27**:2194–2200.

Hales B, Edwards C, Ritchie D, Hall G, Pickup R, Saunders J. (1996). Isolation and identification of methanogen-specific DNA from blanket bog peat by PCR amplification and sequence analysis. *Appl Envir Microbiol* **62**:668–675.

Hall BG. (2013). Building phylogenetic trees from molecular data with MEGA. *Mol Biol Evol* **30**:1229–35.

Holmes AJ, Costello AM, Lidstrom ME, Murrell JC. (1995). Evidence that particulate methane monooxygenase and ammonia monooxygenase may be evolutionarily related. *FEMS Microbiol Lett* **132**:203–8.

Huson DH, Richter DC, Rausch C, Dezulian T, Franz M, Rupp R. (2007). Dendroscope: An interactive viewer for large phylogenetic trees. *BMC Bioinformatics* **8**:460.

Lozupone C, Hamady M, Knight R. (2006). UniFrac - An online tool for comparing microbial community diversity in a phylogenetic context. *BMC Bioinformatics* **7**:371.

Luesken FA, Zhu B, van Alen TA, Butler MK, Diaz MR, Song B, *et al.* (2011). pmoA Primers for detection of anaerobic methanotrophs. *Appl Environ Microbiol* **77**:3877–80.

McKew BA, Coulon F, Osborn AM, Timmis KN, McGenity TJ. (2007). Determining the identity and roles of oil-metabolizing marine bacteria from the Thames estuary, UK. *Environ Microbiol* **9**:165–76.

McMurdie PJ, Holmes S. (2013). phyloseq: an R package for reproducible interactive analysis and graphics of microbiome census data. *PLoS One* **8**:e61217.

Oksanen J, Guillaume Blanchet F, Kindt R, Legendre P, Minchin PR, O'Hara RB, *et. al.* (2015). Vegan: Community Ecology Package. http://cran.r-project.org/package=vegan.

Pester M, Friedrich MW, Schink B, Brune A. (2004). pmoA-Based Analysis of Methanotrophs in a Littoral Lake Sediment Reveals a Diverse and Stable Community in a Dynamic Environment. *Appl Environ Microbiol* **70**:3138–3142.

Steinberg LM, Regan JM. (2008). Phylogenetic comparison of the methanogenic communities from an acidic, oligotrophic fen and an anaerobic digester treating municipal wastewater sludge. *Appl Environ Microbiol* **74**:6663–71.

Tamura K, Stecher G, Peterson D, Filipski A, Kumar S. (2013). MEGA6: Molecular Evolutionary Genetics Analysis version 6.0. *Mol Biol Evol* **30**:2725–9.

Yang S, Liebner S, Alawi M, Ebenhöh O, Wagner D. (2014). Taxonomic database and cut-off value for processing mcrA gene 454 pyrosequencing data by MOTHUR. *J Microbiol Methods* **103**:3–5.

Zhang J, Kobert K, Flouri T, Stamatakis A. (2014). PEAR: a fast and accurate Illumina Paired-End reAd mergeR. *Bioinformatics* **30**:614–20.

**Table S1.** Water samples for molecular analysis.

| **Targeted process** | **Water depth (m)** | **Latitude/**  **Longitude** | **Date** | **Filter diameter (mm)/ type** | **Volume filtered (L)** | **Volume per surface area (mL/mm^2^)** |
| --- | --- | --- | --- | --- | --- | --- |
| MO | 30 | 12°N/  92°30W | 14/12/11 | 293/ PC | 32 | 0.47 |
| MO | 180 | 12°N/  92°30W | 14/12/11 | 293/ PC | 791 | 11.47 |
| MO | 1250 | 13°N/  92°30W | 20/12/11 | 293/ PC | 1255 | 18.20 |
| MO | 1130 | 11°N/  92°30W | 27/12/11 | 293/ PC | 221 | 3.20 |
| MO | 1080 | 10°N/  92°30W | 02/01/12 | 293/ PC | 210 | 3.04 |
| MO | 290 | 9°30N/  92°30W | 09/01/12 | 293/ PC | 210 | 3.04 |
| MO | 645 | 9°30N/  92°30W | 09/01/12 | 293/ PC | 330 | 4.78 |
| NDAMO | 235 | 13°25N/  91°23W | 17/01/14 | 293/ PC | 473 | 6.86 |
| MO | 215 | 13°21N/  91°23W | 23/01/14 | 293/ Supor | 603 | 8.74 |
| MO | 200 | 13°21N/  91°23W | 23/01/14 | 293/ Supor | 502 | 7.28 |
| NDAMO | 332 | 13°21N/  91°23W | 29/01/14 | 293/ Supor | 459 | 6.65 |
| MO | 226 | 13°16N/  91°08W | 30/01/14 | 47/ Supor | 5 | 2.88 |
| MO; NDAMO | 228 | 13°16N/  91°08W | 02/02/14 | 293/ Supor | 1146 | 16.62 |
| NDAMO | 256 | 13°16N/  91°08W | 02/02/14 | 47/ Supor | 3 | 1.73 |
| NDAMO | 264 | 13°16N/  91°08W | 02/02/14 | 47/ Supor | 3 | 1.73 |

PC stands for polycarbonate filters; MO for aerobic methane oxidation and NDAMO for nitrite-dependent anaerobic methane oxidation.

**Table S2.** Identity, relative abundance (%) and diversity of the *mcrA* OTUs from seabed sediments (inshore samples).

|  |  |  | **% Relative abundance of sequences in each sample** | | | | |
| --- | --- | --- | --- | --- | --- | --- | --- |
| **OTU** | **Closest relative (Acc. Number)** | **ID(%)** | **222m** | **342m** | **657m** | **650m** | **550m** |
| ETNP_MG1 | *Methanococcoides* sp. MO-MCD  **(**AB598292) | 97 | 7.64 | 8.82 | 4.38 | 2.14 | 12.05 |
| ETNP_MG2 | *Methanococcoides methylutens* (U22235) | 96 | 6.24 | 11.51 | 10.99 | 9.78 | 11.48 |
| ETNP_MG3 | uncult. archaeon **(**HQ635732) | 94 | 0.00 | 1.54 | 0.00 | 0.00 | 0.00 |
| ETNP_MG4 | *Methanococcoides* sp. PM2 (HE862415) | 87 | 2.91 | 0.00 | 0.00 | 0.81 | 0.00 |
| ETNP_MG5 | uncult. archaeon **(**EF202799) | 95 | 0.00 | 0.00 | 0.00 | 1.08 | 0.00 |
| ETNP_MG6 | uncult. archaeon **(**EU302048) | 98 | 0.00 | 0.00 | 0.00 | 0.67 | 0.00 |
| ETNP_MG7 | *Methanococcoides* sp. PM2 (HE862415) | 87 | 0.00 | 0.03 | 0.00 | 0.34 | 0.00 |
| ETNP_MG8 | *Methanococcoides* sp. PM2 (HE862415) | 88 | 0.00 | 0.39 | 0.20 | 0.00 | 0.00 |
| ETNP_MG9 | *Methanococcoides* sp. MO-MCD  **(**AB598292) | 88 | 0.00 | 0.23 | 0.00 | 0.00 | 0.00 |
| ETNP_MG10 | *Methanococcoides methylutens* (U22235) | 84 | 0.12 | 0.00 | 0.00 | 0.00 | 0.00 |
| ETNP_MG11 | uncult. archaeon **(**FJ264879) | 98 | 0.00 | 0.00 | 0.00 | 0.16 | 0.00 |
| ETNP_MG12 | Methanococcoides methylutens (U22235) | 95 | 0.02 | 0.01 | 0.66 | 0.00 | 0.02 |
| ETNP_MG13 | *Methanosarcina* horonobensis (AB288266) | 93 | 0.00 | 0.00 | 0.00 | 0.14 | 0.00 |
| ETNP_MG14 | *Methanocella arvoryzae* MRE50 (AM114193) | 90 | 0.00 | 0.00 | 0.00 | 0.06 | 0.00 |
| ETNP_MG15 | *Methanococcoides methylutens* (U22235) | 99 | 0.42 | 2.84 | 1.15 | 0.69 | 0.03 |
| ETNP_MG16 | *Methanococcoides* sp. PM2 (HE862415) | 87 | 0.24 | 0.17 | 0.00 | 0.01 | 0.00 |
| **Sum** |  |  | **17.60** | **25.54** | **17.39** | **15.90** | **23.58** |
| **Shannon** |  |  | 1.22 | 1.29 | 0.99 | 1.37 | 0.71 |
| **Simpson** |  |  | 0.66 | 0.66 | 0.53 | 0.59 | 0.50 |

**Table S3**. Identity, relevant abundance (%) and diversity of the *pmoA* (offshore and inshore water column samples) OTUs.

| **OTU** | **Closest relative (Acc. Number)** | **ID**  **(%)** | **% Relative abundance of sequences in each sample** | | | | | | |
| --- | --- | --- | --- | --- | --- | --- | --- | --- | --- |
| **MO; Offshore** | | | **30m** | **180m** | **1250m** | **1130m** | **1080m** | **290m** | **645m** |
| ETNP_  Offshore_MO1 | uncult. bacter. (LK021570) | 100 | 1.93 | 0.13 | 9.75 | 5.85 | 8.30 | 17.20 | 0.98 |
| ETNP_  Offshore_MO2 | uncult. bacter. (EU444843) | 100 | 0.00 | 0.94 | 13.32 | 11.92 | 8.14 | 12.88 | 0.00 |
| ETNP_  Offshore_MO3 | uncult. bacter. (LK021570) | 99 | 0.02 | 0.05 | 0.35 | 0.16 | 0.47 | 2.66 | 0.08 |
| ETNP_  Offshore_MO4 | uncult. bacter.  (FJ858373) | 100 | 0.00 | 0.03 | 0.15 | 0.48 | 0.34 | 2.95 | 0.00 |
| ETNP_  Offshore_MO5 | uncult. bacter. (LK021570) | 99 | 0.00 | 0.00 | 0.08 | 0.03 | 0.11 | 0.45 | 0.00 |
| ETNP_  Offshore_MO6 | uncult. bacter.  (AB276028) | 98 | 0.00 | 0.05 | 0.08 | 0.02 | 0.00 | 0.08 | 0.00 |
| **Sum** |  |  | **1.95** | **1.19** | **23.73** | **18.46** | **17.37** | **36.23** | **1.06** |
| **Shannon** | |  | 0.05 | 0.79 | 0.82 | 0.80 | 0.91 | 1.19 | 0.27 |
| **Simpson** | |  | 0.02 | 0.37 | 0.52 | 0.48 | 0.55 | 0.64 | 0.14 |
| **MO; Inshore** | |  | **200m** | **215m** | **226m** | **228m** |  |  |  |
| ETNP_  Inshore_MO1 | uncult. bacter. (AB276025) | 100 | 20.22 | 8.02 | 22.53 | 38.65 |  |  |  |
| ETNP_  Inshore_MO2 | uncult. bacter. (AB276028) | 99 | 2.67 | 0.78 | 0.01 | 1.96 |  |  |  |
| ETNP_  Inshore_MO3 | uncult. bacter.  (AB276027) | 97 | 1.77 | 0.49 | 0.88 | 1.84 |  |  |  |
| ETNP_  Inshore_MO4 | uncult. bacter.  (FJ858373) | 100 | 0.00 | 0.00 | 0.01 | 0.01 |  |  |  |
| ETNP_  Inshore_MO5 | uncult. bacter.  (AB276025) | 100 | 0.11 | 0.01 | 0.02 | 0.00 |  |  |  |
| ETNP_  Inshore_MO6 | uncult. bacter.  (FJ858374) | 97 | 0.00 | 0.00 | 0.00 | 0.00 |  |  |  |
| **Sum** |  |  | **24.79** | **9.30** | **23.44** | **42.47** |  |  |  |
| **Shannon** | |  | 0.62 | 0.50 | 0.17 | 0.37 |  |  |  |
| **Simpson** | |  | 0.32 | 0.25 | 0.07 | 0.17 |  |  |  |
| **NDAMO; Inshore** | |  | **235m** | **332m** | **228m** | **256m** | **264m** |  |  |
| ETNP_  NDAMO1 | uncult. bacter.  (KF742448) | 98 | 0.00 | 1.4×10^-4^ | 1.4×10^-4^ | 1.4×10^-4^ | 2.8×10^-4^ |  |  |
| ETNP_  NDAMO2 | uncult. bacter.  (KF742448) | 90 | 31.18 | 26.01 | 9.97 | 16.69 | 16.15 |  |  |
| **Sum** |  |  | **31.18** | **26.01** | **9.97** | **16.69** | **16.15** |  |  |
| **Shannon** |  |  | 0 | 0.00007 | 0.0002 | 0.0001 | 0.0002 |  |  |
| **Simpson** |  |  | 0 | 0.00001 | 0.00003 | 0.00002 | 0.00004 |  |  |

Offshore samples were collected during the first cruise (D373), from 13°N, at approximately 140km, to 9°30N, at approximately 500km off the west coast of Guatemala. Inshore samples were collected during the second cruise (JC097), some 58-70km off the Guatemalan coast.

**Figure S1.** a) Map of the offshore and inshore sampling sites. b) Oxygen (µmol L^-1^), c) methane (nmol L^-1^) and d) nitrite (µmol L^-1^) concentrations changing with depth across the sampling locations. Black dots indicate the exact locations where measurements were made.

**Figure S2.** Sampling depths for methane oxidation (aerobic and N-DAMO), shown in relation to the oxygen, methane, nitrous oxide and nitrite profiles.

**Figure S3.** Nitrite, nitrate and sulphate sediment porewater concentrations.
