# Supplementary figures and images for: Origin and fate of methane in the Eastern Tropical North Pacific oxygen minimum zone
Source: ISME J. 2017 Feb 28;11(6):1386–99. doi: 10.1038/ismej.2017.6 (PMC5437358; doi:10.1038/ismej.2017.6)

Figure S1

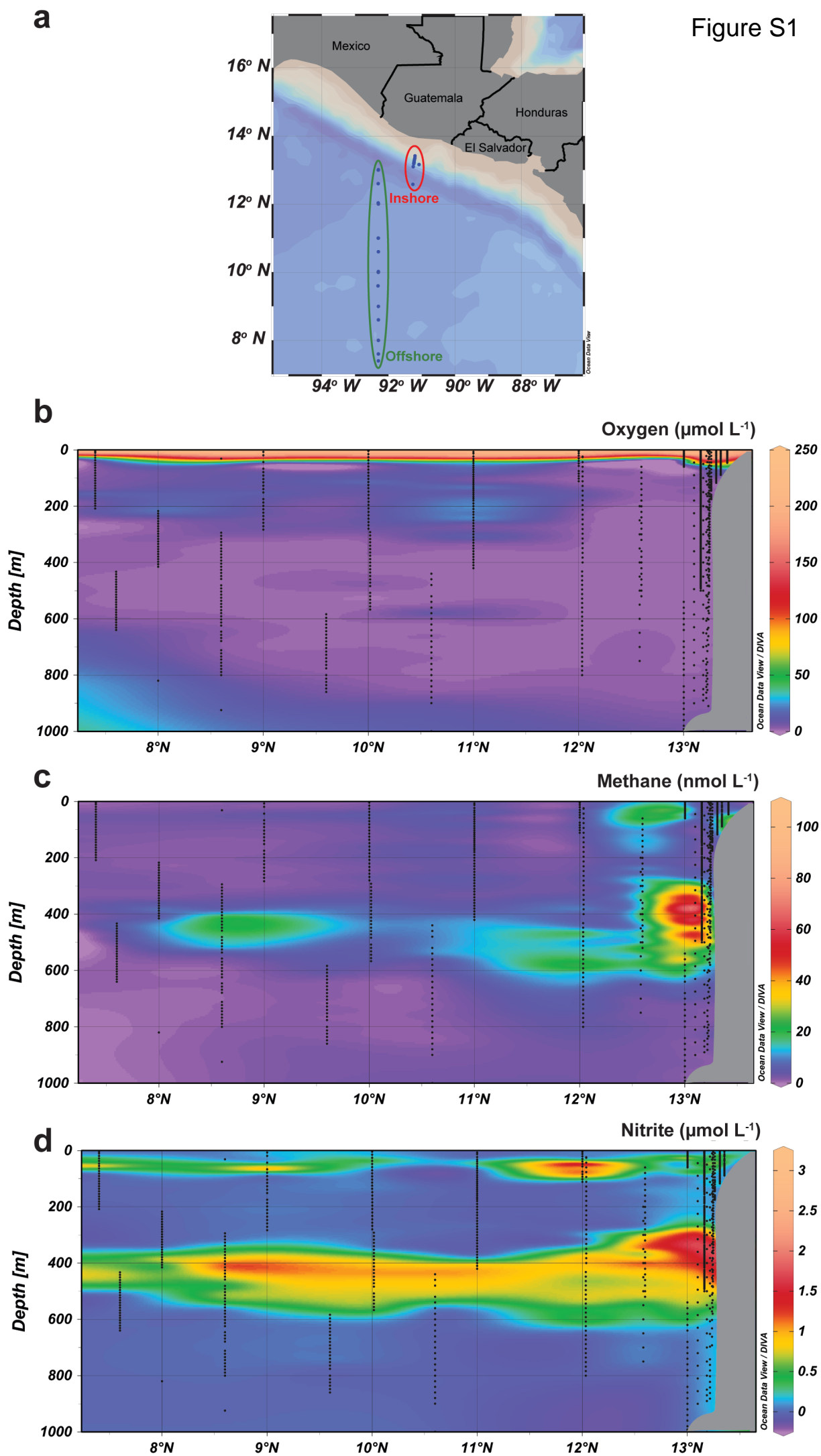

Supplement: Supplementary Figure S1 [file ismej20176x2.pdf]

Figure S2

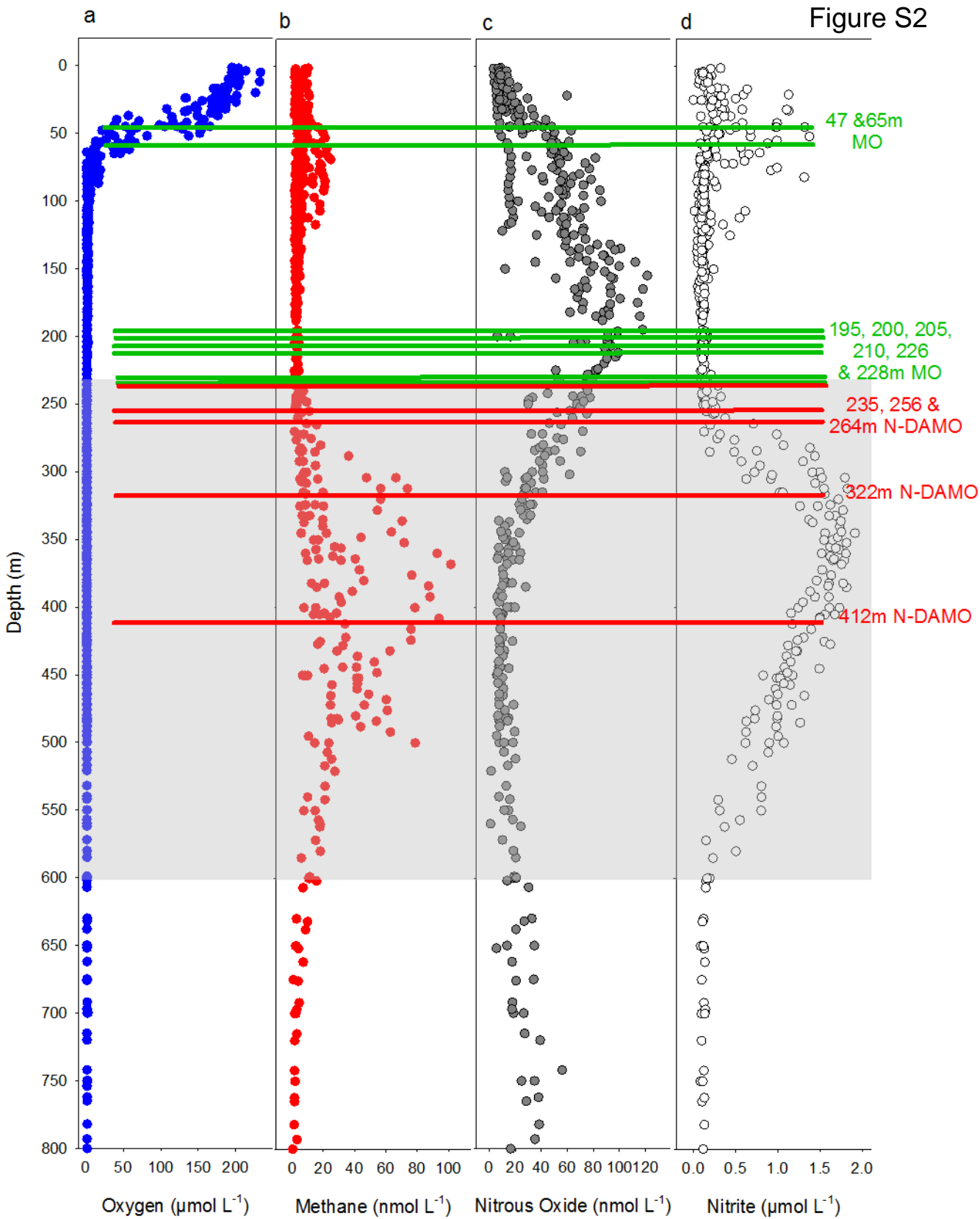

Supplement: Supplementary Figure S2 [file ismej20176x3.pdf]

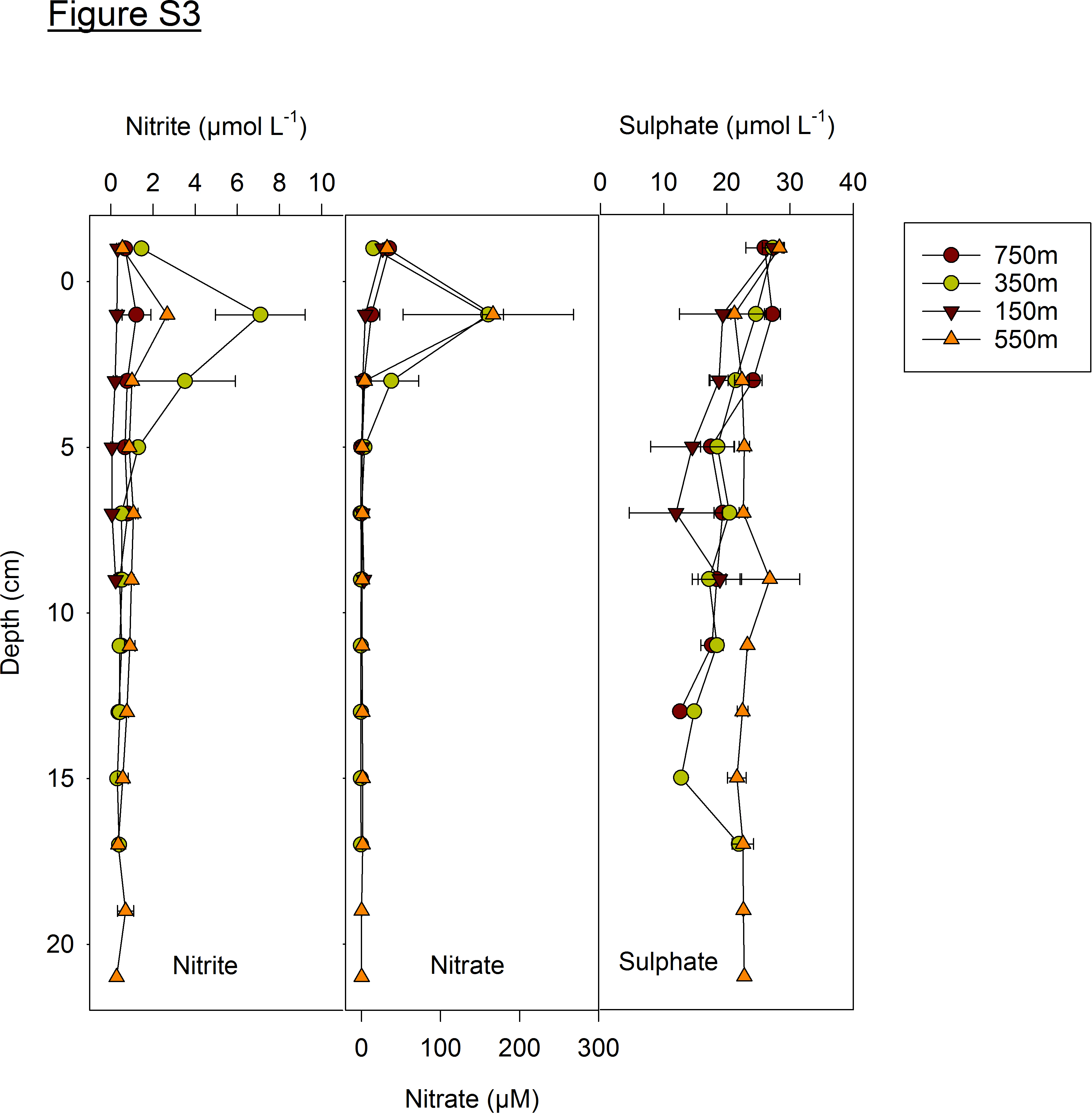

Supplement: Supplementary Figure S3 [file ismej20176x4.tif]
